# Pancreatitis Preceding the Diagnosis of IBD in Children: A Retrospective Observational Study

**DOI:** 10.3390/children12091138

**Published:** 2025-08-28

**Authors:** Lorenzo D’Antonio, Valerio Balassone, Federico Alghisi, Chiara Imondi, Francesca Rea, Erminia Romeo, Giulia Angelino, Sabrina Cardile, Daniela Knafelz, Fiammetta Bracci, Paola De Angelis, Simona Faraci

**Affiliations:** 1 Gastroenterology, Endoscopy and Surgery Unit, Bambino Gesù Children’s Hospital, IRCCS, 00165 Rome, Italy; 2 Pediatrics Section, Department of Translational Medical Sciences, University of Naples Federico II, 80131 Naples, Italy; 3 Pediatric Pulmonology & Cystic Fibrosis Unit, Bambino Gesù Children’s Hospital, IRCSS, 00165 Rome, Italy

**Keywords:** pancreatitis, inflammatory bowel disease, extraintestinal manifestations

## Abstract

**Highlights:**

**What are the main findings?**

- This study identifies pancreatitis as a possible early manifestation of IBD in children.
- Fecal calprotectin was elevated in all tested pediatric patients with pancreatitis who were later diagnosed with IBD.

**What is the implication of the main finding?**

- IBD should be considered in the differential diagnosis of pediatric pancreatitis, particularly in idiopathic cases.
- Fecal calprotectin testing should be included in the diagnostic workup for pediatric pancreatitis

**Abstract:**

**Background**: Pancreatic involvement in inflammatory bowel diseases (IBD) is relatively common and includes a range of conditions, such as acute pancreatitis (AP), chronic pancreatitis (CP), autoimmune pancreatitis (AIP), and pancreatic exocrine insufficiency (PEI). However, pancreatitis as a precursor to IBD is not well understood and is rarely reported. **Objectives**: This study investigates the occurrence, etiology, severity, and recurrence patterns of acute pancreatitis (AP) prior to IBD diagnosis in pediatric patients, with the aim of improving early recognition and diagnostic approaches. **Methods**: This retrospective observational study was conducted between January 2019 and December 2023 at a tertiary pediatric center, including patients who developed pancreatitis prior to an IBD diagnosis. Demographic information, clinical presentation, laboratory findings, imaging results, fecal calprotectin levels, radiological tests, blood tests, and endoscopic findings were collected. **Results**: Among 312 pediatric IBD patients (99 with Crohn’s disease (CD), 162 with ulcerative colitis (UC), 7 unclassified, and 44 with very early-onset IBD [VEO-IBD]), 11 (3.5%) had pancreatitis preceding the IBD diagnosis. All the patients showed elevated fecal calprotectin levels, and endoscopy confirmed IBD (four with CD, seven with UC). The median time from the onset of pancreatitis to the IBD diagnosis was 77 weeks (range 0–366 weeks). **Conclusions**: This study supports the hypothesis that pancreatitis may precede the diagnosis of IBD in some cases, acting as an early extraintestinal manifestation, as previously reported in adults. IBD should be considered in the differential diagnosis of pediatric pancreatitis, particularly in idiopathic cases. Fecal calprotectin testing should be included in the diagnostic workup for pediatric pancreatitis at both initial presentation and during follow-up. Further research is needed to better understand the mechanisms underlying this extraintestinal manifestation.

## 1. Introduction

Pancreatic involvement in inflammatory bowel disease (IBD) is relatively common and includes a heterogeneous group of conditions, such as acute pancreatitis (AP), chronic pancreatitis (CP), autoimmune pancreatitis (AIP), and pancreatic exocrine insufficiency (PEI).

Pancreatic involvement is well recognized as an extraintestinal manifestation of IBD, along with involvement of other organs, such as the liver, lungs, skin, and eyes [1]. The spectrum of pancreatic manifestations in IBD can range from mild, self-limiting conditions or severe diseases to asymptomatic changes detected through imaging and laboratory tests.

The literature well describes the association of acute pancreatitis or hyperenzinemia as potential side effects of medications, particularly 5-aminosalicylic acid (5ASA) and thiopurines [2].

Although AP or CP associated with IBD are considered rare, asymptomatic PEI, pancreatic duct abnormalities, and hyperenzinemia are observed in up to 18% of patients with IBD [3]. Moreover, Heikius et al. reported that high levels of serum amylase and lipase were associated with extensive colonic disease and high histological activity [4].

If it is well established that patients with IBD have an increased risk of developing pancreatitis [5]; similarly, individuals with CP have a higher likelihood of developing IBD with an overall incidence 10.3 times greater [6]. Despite being increasingly recognized in adult clinical practice, the association between IBD and pancreatitis is underexplored in pediatric age.

Few studies have reported pancreatitis as an initial manifestation of IBD, and most of these have focused on adult populations. One study in an Israeli cohort reported that idiopathic pancreatitis, occurring before the diagnosis of IBD, is more frequent in pediatric patients than in adults with a prevalence of 2.2% [7].

The role of pancreatitis as a potential early indicator of IBD, particularly in pediatric populations, is still not well understood. Recognizing pancreatitis as a precursor to IBD could lead to earlier diagnosis and intervention, potentially improving the long-term management of these patients.

This study aims to describe pancreatitis as an early extraintestinal manifestation and precursor of IBD in our case series of pediatric patients.

## 2. Materials and Methods

**Figure 1:**
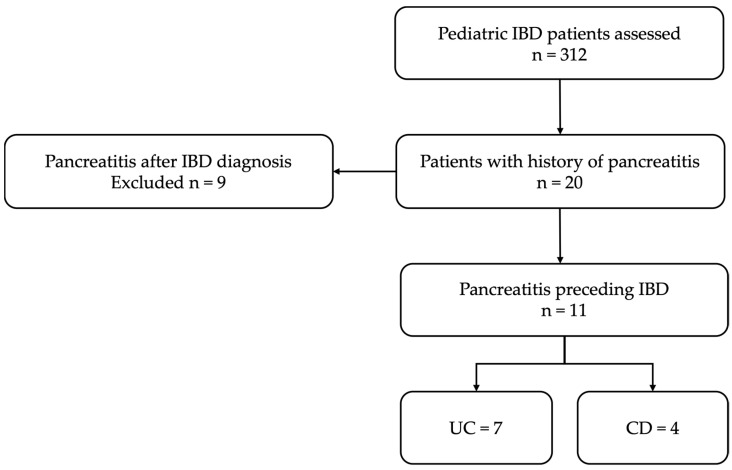
Flow diagram summarizing the case selection process for the study.

This is a retrospective observational study conducted at a tertiary pediatric center. The study was conducted between January 2019 and December 2023, a period chosen to ensure completeness and uniformity of electronic medical records. Consecutive pediatric patients, who were diagnosed with unexplained pancreatitis followed by IBD were included. The inclusion and exclusion criteria are summarized in Table 1. The case selection process for the study has been summarized in the flow diagram shown in Figure 1.

Data were obtained from the electronic medical records of patients who met the inclusion criteria. Demographic information, clinical presentation, laboratory results, and imaging findings were collected. Specific clinical features of pancreatitis, such as abdominal pain, nausea, vomiting, and changes in serum amylase and lipase levels, were recorded. Radiological findings from abdominal ultrasound, computed tomography (CT), and magnetic resonance imaging (MRI) were reviewed for signs of pancreatic inflammation or structural changes. Blood tests, including complete blood count (CBC), liver function tests (LFTs), inflammatory markers (CRP, ESR), and pancreatic function tests (amylase and lipase) were reviewed. Genetic testing, ERCP, MRCP, and IgG4, were evaluated in case of recurrent and chronic pancreatitis in relation to clinical history [8].

Definition of acute pancreatitis (AP) was based on at least two of the following: (a) Typical abdominal pain; (b) serum amylase and/or lipase > 3× upper limit of normal (ULN); (c) imaging findings compatible with AP.

Severity of pancreatitis was graded retrospectively according to the Revised Atlanta Classification into mild, moderately, and severe, based on presence and duration of organ failure and/or local complications [9].

Idiopathic pancreatitis was defined as cases where biliary, anatomic, infectious, metabolic, autoimmune, and drug-induced etiologies were excluded through appropriate workup, including viral serologies, metabolic panel, imaging, and genetic testing when indicated.

Fecal calprotectin level was measured during the episode of pancreatitis and at subsequent follow-up visits. Fecal calprotectin is a well-established biomarker for intestinal inflammation, and its elevation has been shown to correlate with disease activity in IBD [10]. Endoscopic evaluations with biopsies from inflamed and uninflamed segments (esophagogastroduodenoscopy, ileocolonoscopy, and capsule endoscopy) were performed for diagnosis and classification of associated IBD.

The diagnosis of IBD was based on clinical, endoscopic, histological, and radiological criteria, in accordance with ECCO guidelines [11].

## 3. Results

A total of 312 pediatric IBD patients were identified; 99 were diagnosed with CD (31.7%), 162 with UC (52%), 7 with unclassified IBD (2.2%), 44 (14.1%) with very early-onset IBD (VEO-IBD).

Among these 312 patients, 11 (3.5%) had pancreatitis preceding the diagnosis of IBD. Demographic characteristics, clinical data, and laboratory tests of these 11 patients are presented in Table 2 and Table 3.

**Figure 2:**
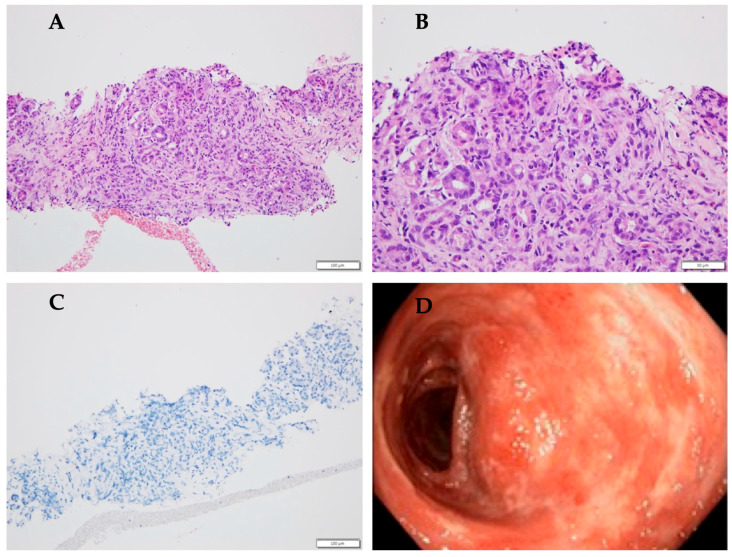
Histological and endoscopic findings in a patient with idiopathic pancreatitis and subsequent diagnosis of ulcerative colitis. (**A**) Histological section of pancreatic parenchyma (hematoxylin–eosin staining, low magnification, scale bar = 100 µm) showing preserved lobular structure with diffuse interstitial inflammatory infiltration. (**B**) Same pancreatic biopsy as in panel A at higher magnification (scale bar = 50 µm). (**C**) MUM-staining negative for IgG4 (scale bar = 100 µm). (**D**) High-definition white-light colonoscopy view of the descending colon showing erythematous, friable mucosa with loss of vascular pattern and confluent superficial ulcerations.

The median age at diagnosis of pancreatitis was 11.5 years. The group consisted of six male and five female patients. All 11 patients presented with symptoms of pancreatitis, including abdominal pain, with or without nausea and vomiting. Laboratory tests showed mild to moderate elevation in serum amylase and/or lipase levels during the episode of pancreatitis (>3xULN), consistent with the diagnosis of acute pancreatitis. In one patient, the elevation of pancreatic enzymes was <3xULN and the diagnosis was based on imaging and clinical presentation. Six patients (54.5%) experienced more than one episode. The exact dates of the episodes were available for four patients, from whom a median time to recurrence emerged of 31 weeks (range: 5–100). Two of them were diagnosed with chronic pancreatitis. Due to multiple episodes of pancreatitis, four patients underwent genetic testing, revealing non-causative mutations in two of them. Additionally, two patients underwent ERCP with sphincterotomy, and one required stent placement due to pancreatic duct dilation. IgG4 levels were normal in all tested patients. A patient with suspected AIP due to enlargement of the pancreatic head underwent an endoscopic ultrasound-guided biopsy, which revealed inflammation cells. This patient was diagnosed with UC 2 years later (Figure 2).

The median time between the first episode of pancreatitis and the subsequent diagnosis of IBD was 77 weeks (range 0–366 weeks), highlighting potential delays in IBD recognition of IBD in some cases. As is well known, extraintestinal manifestations can occur in up to 24% of patients with IBD before the onset of intestinal symptoms [1]. Notably, two patients had a diagnosis of IBD established immediately after the episode of pancreatitis, suggesting that the onset of IBD symptoms may occur shortly after, or even years following, the initial pancreatitis episode.

At our center, during the follow-up of patients with idiopathic acute pancreatitis or chronic pancreatitis, a fecal calprotectin test is often incorporated into the diagnostic workup, considering the association with IBD. For this reason, fecal calprotectin level was tested in nine patients with pancreatitis preceding IBD, and in all cases, the levels exceeded the normal threshold, with values greater than 100 mcg/g [10], suggesting significant intestinal inflammation. Endoscopy confirmed the diagnosis of IBD in all patients: four were diagnosed with CD and seven with UC. Among these patients, only one had duodenal involvement. This finding is inconsistent with the literature, where pancreatic manifestations are considered more frequent in patients with CD than in those with UC.

## 4. Discussion

There is limited data on the incidence of AP in pediatric patients with IBD. A retrospective analysis from 2021 reviewed 1538 children diagnosed with IBD and found that 5% had history of AP, and 5.7% had asymptomatic hyperamylasemia and hyperlipasemia. Among these patients, idiopathic pancreatic involvement was the most common (57%), followed by drug-induced involvement (37%) [12]. Similarly, a Polish study analyzing 101 children with IBD (79 with UC and 22 with CD) reported AP occurring in 4.5% of children with CD and 5.1% of those with UC [13]. Unlike pediatric cases, AP in adults is more commonly associated with CD than UC. Although reported incidence rates of AP in IBD patients vary across studies, there is consensus that its occurrence is higher than in the general population.

A study by Weber et al. tracked 852 CD patients over a decade and found that 12 developed AP, resulting in a 1.4% incidence rate [14]. Moreover, in adults, idiopathic pancreatitis is rare with only 8% of 48 cases classified as idiopathic in a study by Moolsintong et al. [15].

There is limited evidence about the association between IBD and CP, but a French study reported the presence of CP in IBD in 1.2–1.5% of the cases, varying according with the diagnostic technique [16]. Diagnostic criteria for AP, ARP, and CP in children are reported in Table 4 [17].

The development of AP in IBD is multifactorial. In adults, gallstones, alcoholism, and medications are the most common causes of AP in IBD, while post-endoscopic retrograde cholangiopancreatography (ERCP), immune system dysfunction, duodenal involvement, hypercalcemia, and hypertriglyceridemia are less common causes [3]. In pediatric patients, gallstones and alcohol consumption are rare, whereas upper gastrointestinal and duodenal involvement are more frequently observed [18]. In our study, we excluded patients with drug-induced pancreatitis, focusing on those with spontaneous pancreatic involvement as part of their underlying IBD. The phenotype of disease was classified using Paris classification [19]. Among the 11 patients, 2 patients of the 4 with CD had upper gastroesophageal involvement, but only 1 case had duodenal involvement. The findings of this study support the hypothesis that pancreatitis can precede the onset of IBD in some pediatric patients, acting as an early extraintestinal manifestation of the disease. This observation has significant implications for the diagnostic approach to children with unexplained pancreatitis. In cases where pancreatitis is the primary presenting feature, IBD should be included in the differential diagnosis, particularly in patients with risk factors for autoimmune diseases.

In adults, ECCO Collaborative Network For Exceptionally Rare case reports project (ECCO-CONFER) collected a large international cohort of patients with concomitant AIP-IBD, and most of them have type 2 AIP and colonic IBD, suggesting a strong correlation between these two autoimmune diseases. Therefore, in pediatric patients with IBD, pancreatitis could actually be considered as type 2 autoimmune pancreatitis [8].

Early recognition of this association could facilitate the timely diagnosis and management of IBD, potentially improving long-term outcomes for these patients. Fecal calprotectin may be a useful tool in this context. In fact, in this study, all tested patients with pancreatitis preceding IBD had elevated fecal calprotectin levels, suggesting that this test could be used to identify patients at higher risk of developing IBD. Elevation in fecal calprotectin, combined with clinical suspicion of IBD, should prompt further diagnostic testing, including endoscopy and imaging studies.

This study has several limitations, including its retrospective design and the relatively small sample size. The absence of a control group and the small sample size preclude definitive conclusions. We acknowledge that no statistical comparison with general pediatric populations was performed, limiting causal inference.

Larger, multicenter studies are needed to confirm these findings and to investigate the underlying mechanisms linking pancreatitis and IBD.

## 5. Conclusions

Our study highlights pancreatitis as a potentially early manifestation of IBD in pediatric patients. Given the potential for early recognition, we suggest that pancreatitis in pediatric patients, particularly in unexplained cases, should raise suspicion for IBD, and that fecal calprotectin testing should be incorporated into the diagnostic workup. This may lead to earlier detection and treatment of IBD, potentially improving outcomes for these patients. Given the low incidence and retrospective nature, these findings should be interpreted with caution, and larger multicenter controlled studies are needed. Further studies are required to explore the underlying mechanisms of pancreatitis in IBD and to establish evidence-based guidelines for the management of these complex cases.

## Figures and Tables

**Table 1:** Inclusion and exclusion criteria for case selection.

Inclusion Criteria	Exclusion Criteria
Age < 18 years at the diagnosis of pancreatitis	Secondary causes of pancreatitis, (biliary disease, infections, or medication, and malformations)
Diagnosis of acute pancreatitis according to the Revised Atlanta Classification (see below) prior to diagnosis of IBD	
Available clinical, laboratory, imaging, and follow-up data	

**Table 2:** Clinical and biochemical characteristics of patients with acute pancreatitis preceding IBD diagnosis.

Patient N.	Age, y	Type of IBD	N. AP Episodes Preceding IBD	Recurrence Time (Wks Between First and Second AP)	Lag Time Between AP and IBD, wk	Lipase Levels in AP	Amylase Levels in AP
1	15	UC	2	N/A	78.43	>4000	-
2	9	CD	1	-	0.00	1441	520
3	12	UC	1	-	0.00	92	81
4	12	CD	4	8	47.71	900	500
5	14	UC	2	100	100.00	2420	363
6	18	UC	2	12	100.29	1700	700
7	6	UC	1	1	4.43	770	746
8	11	CD	1	1	8.71	297	78
9	10	CD	3	N/A	221.86	-	-
10	11	UC	1	1	17.43	2500	-
11	12	UC	3	5	165.29	260	349

IBD, Inflammatory Bowel Disease; AP, Acute Pancreatitis; UC, Ulcerative Colitis; CD, Crohn Disease; N/A not available.

**Table 3:** Clinical, biochemical, endoscopic, genetic, and therapeutic findings in patients with acute pancreatitis preceding IBD diagnosis.

Patient N.	Fecal Calprotectin at Diagnosis	Paris Classification	Pancreatic Insufficiency	Chronic Pancreatitis	Atlanta Classification	Necessity of ERCP	Genetic for Pancreatitis	Biologics for Induction °
1	no	E1	yes	yes	mild	no	CASR heterozygosis *	no
2	370	L4aL3	no	no	mild	no	no	no
3	1196	E2	no	no	mild	no	no	no
4	426	L3	no	yes	moderate	yes + sphincterotomy + stenting	negative	no
5	>3500	E4	no	no	mild	no	no	yes, infliximab
6	120	E1	no	no	mild	no	CFTR heterozygosis	no
7	no	E4	no	no	mild	no	no	yes, infliximab
8	232	L4aL3	no	no	mild	no	no	no
9	>250	L1	no	no	mild	no	no	no
10	840	E2	no	no	mild	no	no	no
11	>500	E1	no	no	moderate	yes + sphincterotomy	negative	no

ERCP, endoscopic retrograde cholangiopancreatography; ° indicates whether the IBD severity and classification warranted starting treatment with a biologic agent from the outset, specifying the drug used. * The patient presented the variant c.1661G>A in heterozygosis in the CASR gene, currently classified as a variant of uncertain significance (VUS) with undefined functional and clinical impact.

**Table 4:** Diagnostic criteria for AP, ARP, CP.

AP (At Least 2 of 3)	ARP	CP (At Least 1 of 3)
Typical abdominal pain	At least two episodes of AP	Abdominal pain of pancreatic origin in the context of imaging compatible with chronic pancreatic damage
Serum amylase and/or lipase > 3xULN	Complete resolution in the inter-critical periods	Exocrine pancreatic insufficiency with imaging abnormalities suggestive of CP
Imaging findings compatible with AP		Endocrine pancreatic insufficiency with imaging abnormalities compatible with CP

## Data Availability

The raw data supporting the conclusions of this article will be made available by the authors on request.
